# Intra-operative real time intracranial subarachnoid haemorrhage during glial tumour resection: A case report

**DOI:** 10.1186/1757-1626-1-306

**Published:** 2008-11-11

**Authors:** Tayfun Hakan, Cezmi Çağrı Türk, Hasan Çelik

**Affiliations:** 1 Haydarpasa Numune Teaching and Research Hospital, Clinic of Neurosurgery, Istanbul, Turkey

## Abstract

Glial tumours associated with subarachnoid haemorrhage are very rare. A 64-year-old woman admitted with a history of 3 weeks seizures and a left sided hemiparesis and dysphasia. The magnetic resonance disclosed heterogeneously enhancing a right temporal mass. During surgery, suddenly an abrupt and extensive swelling had occurred both in tumour and the brain tissue. The surgery was completed with a gross total tumour resection together with a partial temporal lobectomy. Postoperative computerized tomography demonstrated a massive subarachnoid hemorrhage (SAH). A cerebral Magnetic Resonance (MR) angiography showed neither an aneurysm nor arteriovenous malformation. Coincidence of an intracerebral tumour and subarachnoid haemorrhage would be devastating.

## Background

Glial tumours associated with intracranial haemorrhage are seldom but well known pathologies. As regarding the coexistences of glial tumours and haemorrhages, all the reported cases are interested in either preoperative or postoperative conditions [1,2]. To the best of our knowledge, we report the first case of a real time subarachnoid haemorrhage (SAH) confirmed by computerized tomography (CT) during intracranial glial tumour resection in a female patient.

## Case report

**Figure 1:**
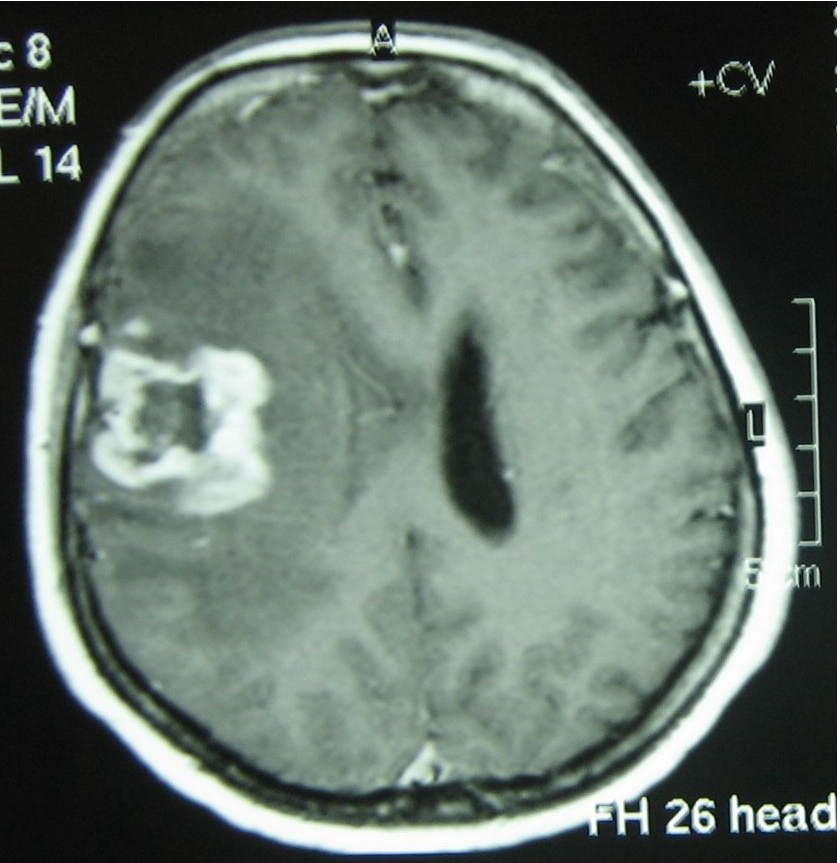
**Post contrast magnetic resonance images demonstrates left temporoparietal tumour with heterogenous rim contrast enhancement.** On the centre of the tumour, hypointense area indicates nectrotic component of the tumour. There is also obvious compression to lateral ventricular wall with prominent cerebral oedema.

**Figure 2:**
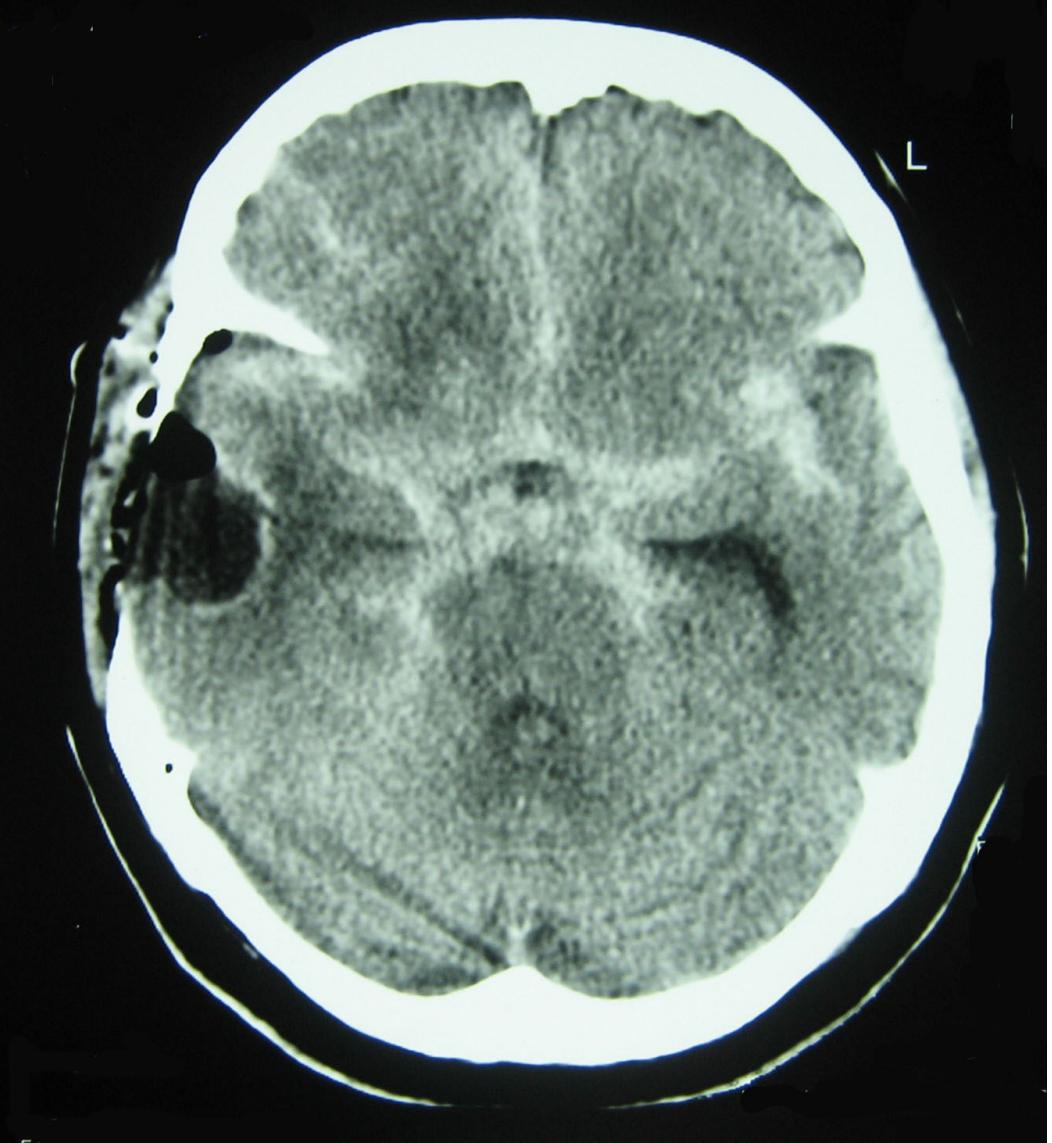
**Post operative CT image shows diffuse subarachnoid haemorrhage in all basal cisterns, bilateral sylvian fissure and interhemispheric fissure.** On the right side postoperative site of tumour removal is seen.

A 64-year-old woman admitted with a history of 3 weeks seizures. Neurologic examination revealed a left sided hemiparesis and dysphasia. The magnetic resonance (MR) images disclosed a right temporal mass with a surrounding oedema. The lesion was heterogeneously enhancing on post contrast images (Figure 1). The tumour had heterogeneous character with necrotic components compatible with glioblastoma. During initial part of the uneventful surgery, suddenly an abrupt and extensive swelling had occurred both in tumour and the brain tissue. The swelling was related neither to venous compromise nor to arterial destruction. No remarkable hypertension or hemodynamic change was detected at that moment. The surgery was completed with gross total tumour resection together with partial temporal lobectomy. By the end of the operation, an immediate cranial CT was achieved and a massive subarachnoid haemorrhage was demonstrated in all subarachnoidal spaces (Figure 2). The preoperative tests including coagulation parameters – bleeding time: 2,5 minutes\Ivy method normal range < 5 min, activated partial tromboplastin time (aPTT): 25 seconds (22,7–31,8), International Normalized Ratio (INR): 0,98 (0,9–1,1) – were within normal ranges that were also true for the postoperative period. A cerebral MR angiography revealed neither an intracranial aneurysm nor vascular malformation. We could not able to perform any digital subtraction angiography (DSA) because of not being available in hospital. Patient was followed in intensive care unit for 35 days after surgery; however she died of pulmonary complications. Autopsy could not been performed for her family did not accepted. The histopathological diagnosis of the tumour was glioblastoma multiforme (GBM).

## Discussion

The most common tumours associated with SAH are pituitary tumours and meningiomas [3,4]. The coexistence of glial tumours and SAH is a rare condition [1,2,5,6]. The occurrence of SAH may be because of a bleeding from a tumour [1,6,7], or an aneurysm [2,5,8] or an invasion of cerebral vessel [9]. Apart from the aneurysms, the cause of the SAH in the preoperative period is mostly related to the location of the tumours along the cerebrospinal pathway near to subarachnoidal space or the ventricles [6,7]. Also, some retiform capillaries have been found responsible in the haemorrhages of astrocytomas and glioblastomas [10].

The previously described SAH and tumour associations are all in preoperative or postoperative period, however the presented case is unique in respect to intraoperative SAH. The intracranial haemorrhages following supratentorial craniotomy is reported remote from surgical site either in supratentorial or infratentorial area [11,12]. However the origins of these bleedings are not well defined yet, changes in the intracranial compartment following the cerebrospinal fluid drainage or displacement of the cerebral\cerebellar tissues was claimed to disturb subcortical veins [11]. Displacement of intracranial structures during surgery, drastic changes both in systolic blood pressure and cerebrospinal fluid pressure, preoperative medications like anti-platelet therapy and tears in bridge veins are thought among the causative factors [11]. Kuroda et al reported [11] subarachnoid haemorrhage in posterior fossa far from the surgical field. The subarachnoid haemorrhage was around the cerebellar fissures and sulci. The cause of the haemorrhage believed was to be related with the tears in the superior vermian vein. All these speculations are mostly for the postoperative bleedings remote from the operative site.

In the presented case, the amount of blood and distribution pattern was far beyond to be explained with localized tears in bridging veins or changes in the dynamics of the intracranial compartment. In our case, coincidental vascular pathologies were suspected because intracranial tumours are rarely associated with intracranial aneurysms. Despite a cerebral MR angiography revealed no vascular pathology neither intracranial aneurysm nor arteriovenous malformation, we believe DSA would be very valuable if it could be performed. Another limitation of our paper is not being able to make an autopsy because of the rejection of the patient's family.

Unrecognized incidental aneurysms may increase the risk of bleeding after surgery for the tumours. In the literature, glial tumours are reported to present with subarachnoid haemorrhage [1], further more invasion of astrocytoma into middle cerebral artery and rupture of the middle cerebral artery on autopsy evaluation was also reported [9].

Theoretical bases for the association of tumour and aneurysms are variable; changes in the hemodynamic factors with increased vascular supply of the tumour [3], hormonal factors [13], unintended injury to vasculature during surgery in case of pituitary tumours [4] and direct invasion of the vasculature [9,14] are reported previously.

It is not hard to acknowledge that coincidence of the tumour resection and subarachnoid haemorrhage would be devastating, as it did in our case. The following case presents a simultaneous subarachnoid bleeding during the initial part of a GBM resection. There was neither venous compromise nor arterial destruction or rapid and extended decompression had provided yet. It can be speculated that the tumour tissue would be compressing an unrecognized coincidental aneurysm. The aneurysm would have been ruptured when the dura opened and the tumour partially resected that provided minimal changes in dynamics of the intracranial compartment. Indeed, invasion of vascular structures by tumour was not concordant with the surgical findings. Whatever reason of the incidence is, what makes this case unique is real time observation of one of the disastrous combination in neurosurgical practice. The presented case, as far as we know, is the first report of simultaneous SAH during GBM excision.

In conclusion, we present the first case of a real time spontaneous SAH arising during intracerebral glioma resection. After excision of the tumour and stabilization of the patient, it should be managed as if it is a primary SAH. In selected cases, performing cerebral DSA in evaluation of the intracerebral malignant tumours locating in the insular region or around sylvian fissure may be warranted to exclude incidental cerebral aneurysm or to verify tumoural implications on the vasculature.

## Consent

Written informed consent was obtained from the patient for publication of this case report and accompanying images.

## Competing interests

The authors declare that they have no competing interests.

## Authors' contributions

TH, HÇ were involved in the patient care, acquisition of data, analysis and interpretation of data, review of literature, drafting and revising the manuscript. CÇT was involved in review of literature, revised the manuscript for important intellectual content. All authors read and approved the final manuscript.
